# Computational Pathology for Breast Cancer and Gynecologic Cancer

**DOI:** 10.3390/cancers15030942

**Published:** 2023-02-02

**Authors:** Ching-Wei Wang, Hikam Muzakky

**Affiliations:** Graduate Institute of Biomedical Engineering, National Taiwan University of Science and Technology, Taipei 106335, Taiwan

Advances in computation pathology have continued at an impressive pace in recent years [1]. New insights into digital pathology, combined with advances in artificial intelligence, computing power, faster networks, and cheaper storage offer more robust forms of cancer prevention, detection, and individualized therapy [2]. Moreover, deep neural networks have transformed the medical imaging field by overcoming multiple imaging challenges [3,4,5,6]. However, analysis of whole slide imaging (WSI) is complicated by extraordinary challenges. WSI is a cutting-edge imaging modality because of its ultra-resolution image (average of 100,000 × 100,000 pixels), presence of coloration patterns from the stain (e.g., hematoxylin and eosin stain), and accessibility of data at multiple magnification levels (e.g., 1×, 10×, 40×). Undoubtedly, a human reader cannot possibly obtain all of the visual information [2].

Furthermore, with the inclusion of digital pathology slides into the pathology work process, advanced algorithm concepts and computer-aided medical diagnostics open up the frontiers of the pathology expert’s vision more than a slide of mounted specimens for microscopic study and facilitate the convergence of knowledge far above human constraints [7]. In order to improve early detection, determine prognosis, and choose the most effective treatments, pathologists can currently use AI to identify specific imaging signals linked to disease processes. As a result, pathologists can provide service to more patients while still providing accurate diagnoses and prognoses [8], while the advantages of AI and digital pathology are growing in many sectors of cancer care and targeted therapies, the focus of this Special Issue is on computational pathology for breast cancer and gynecologic cancer.

Globally, breast cancer is regarded to be the foremost cause of mortality for women [9], and according to the *American Cancer Society*’s report, 43,780 people in the USA were predicted to die from breast cancer in 2022 [10]. Several types of research have been utilized in an attempt to learn more about computational pathology for breast cancer by analyzing histology images together with the patient data they are correlated with. Wang et al. [4] proposed a DL-based method, namely, the soft label fully convolutional network (SL-FCN) to aid breast cancer target therapy by segmenting the human epidermal growth factor receptor 2 (HER2) amplification using fluorescence in situ hybridization (FISH) and dual in situ hybridization (DISH) datasets of metastatic breast cancer. In another study in segmentation, Khalil et al. [11] developed a modified fully convolutional network using histopathological hematoxylin and eosin (H&E) whole-slide images for breast cancer segmentation and assessed the proposed method using H&E- and IHC cytokeratin (AE1/AE3)-stained whole-slide images. This was utilized to create an objective and unbiased reference standard. Apart from segmentation, Wang et al. [12] presented an interactive fully automatic hierarchical registration model which could cross-stain ultra-high resolution of histopathological and immunohistochemical whole-slide images and resolve the major speed bottleneck to help doctors annotate tumor tissue by defining grade and stage of a tumor. Naik et al. [5] demonstrated that biomarker status, as identified by hormone receptors, can be determined by machine learning effectively from cell morphology. ReceptorNet is a multiple-instance learning (MIL)-based method that is proposed for assessing the estrogen receptor status (ERS) from slides that have been H&E-stained. Similarly, on biomarker status prediction, Shamai et al. [6] showed that a convolutional neural network-based framework of whole-slide images (H&E-stained) can predict the expression of specific biomarkers (e.g., PD-L1). The authors of this study make use of a large tissue micro-array repository that contained various respective stains for different biomarkers (e.g., IHC for PD-L1) and a collection of H&E-stained whole-slide images. Additionally, a highly experienced pathologist annotated the expression of PD-L1 for each sample in the datasets.

On the other hand, the World Health Organization (WHO) estimated that cervical cancer affects approximately 300,000 individuals annually, with less developed nations contributing to more than 85% of these deaths in recent years. Cervical cancer, one of today’s most prevalent cancers in women’s health, is diagnosed in one woman per minute [13]. The cervical cancer dataset has been used in various types of research to analyze tissue samples for the study of the disease. Wang et al. [14] introduced a modified FCN for cervical HSILs or higher (SQCC) segmentation of WSIs using Pap smear specimens for use in real-world situations and discovered that the proposed cervical Pap smear diagnosis method could aid with the automatic recognition and quantification of cervical HSILs or higher (SQCC). Zhu et al. [15] proposed an assistive diagnostic, AIATBS, to optimize the assessment of cervical cytological smears using clinical Bethesda system (TBS) standards. Cheng et al. [16] developed a three-stage DL-based model, consisting of a low-resolution model to locate lesion areas at the low-resolution setting, a high-resolution model to recognize the top 10 lesion cells to be fed to the slide-level classification model, and recurrent neural network (RNN)-based technique to integrate the 10 most suspicious input features and yield the result for slide-level prediction.

In addition, ovarian cancer is the second highest frequent gynecologic cancer among women globally [17]. Over 140,000 women die from ovarian cancer worldwide every year, and over 225,000 women are diagnosed with it [18]. To discover more about computational pathology in ovarian cancer, some experiments were conducted utilizing ovarian cancer datasets. Wang et al. [19] proposed a modified FCN technique for predicting the therapeutic efficacy of bevacizumab on patients with ovarian cancer based on histopathological (H&E-stained) whole-slide images, without using any pathologist-provided regionally annotated areas. In a further study, Wang et al. [20] built a modified FCN-based precision oncology system using immunostained tissue microarray (TMA) whole-slide images to determine bevacizumab therapeutic impact in patients with epithelial ovarian cancer (EOC) and peritoneal serous papillary carcinoma (PSPC). In another computational pathology application, Boehm et al. [21] compiled a multi-modality dataset of 444 ovarian cancer patients with the majority of the high-grade serous carcinoma type and found quantitative characteristics on multimodal imaging affiliated with prognosis (e.g., tumor nuclear dimension on H&E staining and omental texture on contrast-enhanced computed tomography).

An analysis of histopathology images involves more than just visual analysis; it also involves data integration from patient clinical information and demographic details [22,23]. Most clinical data appears in unstructured free-text reports, including demographic information, patient test results, patient medical history, and clinical outcomes. The AI-based system role will be crucial in sorting through these many sources of information and supporting pathologists in making the best treatment decisions for patients. The AI-based medication must incorporate clinical data as well as histopathology images, allowing for assessment that exceeds the capabilities of the human brain alone. Rich data resources will aid pathology’s transition from a clinical to an informatics science, with tissue images supplied as one of the information sources [2].

The approaches for AI-based image recognition and its combination with other information sources have developed well within the area of computational pathology toward the level where they might eventually be ready to be converted from the research environment into real clinical laboratories. Despite computational pathology offering a lot of potential avenues, there are still some challenges to overcome [24]. Therefore, we are issuing a call for research papers that utilize innovative approaches for creating future studies to address current challenges in computational pathology for breast cancer and gynecologic cancer. Readers can find more information about publishing research in *Cancers* in the Special Issue “Computational Pathology for Breast Cancer and Gynecologic Cancer”.
